# Supplementary material for: DENDRO: genetic heterogeneity profiling and subclone detection by single-cell RNA sequencing
Source: Genome Biol. 2020 Jan 14;21:10. doi: 10.1186/s13059-019-1922-x (PMC6961311; doi:10.1186/s13059-019-1922-x)
Supplement: Supplementary file 7 — Additional file 7. Review history. [file 13059_2019_1922_MOESM7_ESM.docx]

Review History

**First round of review**

**Reviewer 1**

**Were you able to assess all statistics in the manuscript, including the appropriateness of statistical tests used?**

Yes: I reviewed the equations in the Methods section and supplement.

**Were you able to directly test the methods?**

Yes

**Comments to author:**

The manuscript describes an approach called Dendro for single-cell RNA-seq data. Dendro first characterizes the mutational profiles in each cell, then clusters cells into subclones from which it builds a clonal tree relationship. The relationship can then be used as the basis for further analysis of the transcriptomic aspect of the data. This approach is unique in that it requires no additional data and the case-studies shown are very motivating and would be of broad interest though especially to cancer researchers.

Major Comments:

1. The term 'spike-in procedure' was quite misleading for me since spike-ins have very distinct meaning in transcriptomics. My understanding is that this is a simulation procedure where the structure is simulated rather than the counts themselves. I suggest the authors simply refer to this as a simulation and the explanation given is sufficient.

2. The sentence in line 58-60 regarding limitations to sequencing DNA and RNA in the same cell needs appropriate citations.

3. Equation on line 408, and explanation in line 411-412. If they are from the same clonal group, does that mean the numerator will be smaller than the denominator or just not as large as when they are from different clones? This could be explained more clearly.

4. I appreciate the figure overviews, but some are difficult to follow through. Below are suggestions:

(i)	Figure 1B: Is the top and middle representing both the true underlying data and the bottom is what is observed in the data as a result? Consider adding labels to indicate the distinction or in the legend.

(ii)	Figure 2A: I would label that the mutation profile is simulated and then indicate that bulk is input and then moderated by existing single-cell reference data.

5. QC metrics to retain cells and genes need to be explicitly described. Both the criteria based on mutation, and any typical considerations regarding total number of reads or % mitochondrial reads.

6. Did the authors generate data for this paper on the B16 dataset? If so, where is the data deposited at? For data obtained from publicly available datasets, the GEO/SRA #'s should also be given explicitly.

7. All datasets examined used the Smart-seq full-length protocol. Is it possible to use data from tag based reads (10X, Drop-seq) with Dendro? If so, it would be useful to see as a case-study, or if not then it would be helpful to have a statement on this in the discussion.

8. The software installed and ran as intended. The authors should also add example code on how to make the circular plots like in the paper as that will encourage more users of the package.

Minor Comments:

1. The statement ", that we modeled in DENDRO." In line 65 is a bit odd since DENDRO has not yet been introduced and this is still the background section.

2. Line 395 should say "does not".

3. Clusters on Figure 4b are not indicated as dashed lines.

4. Line 432, the rates, alpha and beta, are estimated empirically as what exactly?

**Reviewer 2**

**Were you able to assess all statistics in the manuscript, including the appropriateness of statistical tests used?**

Yes: I thought that the modeling framework and derivations were sufficiently explained. However, I have certain clarification notes / suggestions in my comments below.

**Were you able to directly test the methods?**

No

**Comments to author:**

The authors have developed a statistical framework, DENDRO, to identify genetically distinct subclones in tumor samples using single-cell (sc-) transcriptomics data. DENDRO accounts for technical noise (induced by errors in reverse transcription, sequencing and mapping) and expression level stochasticity (associated with single-cell level expression bursting) currently inherent in the single-cell transcriptomics library. Once the scRNA-seq-derived mutation profiling is done, DENDRO computes the divergence between pairs of single cells by modeling the conditional mutation frequency (per cell and locus) in a bayesian framework using beta-binomial model (few extensions on this discussed below). With this modeling framework, through simulation and spike-in analyses, the authors show that genetic divergence score is (i) orthogonal to the relative expression level and variation in total expression in genes, and (ii) improves with larger number of mutations profiled, among others. In addition, the authors also show that DENDRO significantly improves over other clustering approach such as hierarchical clustering based on mutation or expression matrix alone and identifies clones better than with scRNA-seq based expression data alone. The authors implemented DENDRO on three real datasets: (i) Implementation on Renal cell carcinoma data with known clonal identity was used as a validation study, with positive affirmation as mentioned above. (ii) subclonal expansion in mouse melanoma model in response to immunotherapy, showing DENDRO identified subclones showing diverging tumor mutational burden and predicted neoantigen load. (iii) primary and lymph-node metastasis in breast cancer samples, showing that DENDRO clusters can identify heterogeneity in otherwise previously mono-labeled breast cancer samples.

Overall the authors have mostly thoroughly explained their approach and model. The implementation of the model, thanks to the R-package, should help users of sc-RNA-seq get genomic mutation profile and clonal detection. The authors have shown that DENDRO performs relatively well with low read coverage and is capable of subclonality detection that are potentially missed by expression level data alone. However, please consider the following comments and questions.

Some comments:

1. Inherently, none of the mutations in the non-coding elements in the genome are considered in this framework/model. While this is a limitation of the assay type itself (scRNA-seq) - and the DENDRO beta-binomial model, by design, only handles mutations along the transcriptome - how robust would the DENDRO clustering itself be to not using non-coding mutational data? In practice, if DENDRO clustering is in fact robust to this (not using non-coding mutational profile), it is also saying that those non-coding mutations do not largely contribute to estimating genetic divergence score, which we know is not necessarily the case. While this (transcriptome-based DENDRO vs 'whole genome mutational profile-based DENDRO') analysis is out of the scope of this paper, the question for the users now would be whether to do scDNA-seq based mutational profiling followed by clonal identification versus just using the scRNA-seq data and DENDRO (which would be cheaper but limited in not having non-exonic mutational profile). Any comment here is appreciated.

2. My understanding is that the performance of clustering (with ARI score) depends on the prior establishment of 'true' tree. In the case of the first implementation (Renal cell carcinoma samples) the clustering was largely separating primary cells from metastatic cells (line 199). However, in the case of the third implementation (primary and lymph-node metastasis in breast cancer samples), DENDRO separates BC03 cells from BC09 cells - and not primary versus metastatic cells - with 100% accuracy. This is not to say the primary versus metastatic split is universal, but, is it not that the ARI is 100% in this case because the 'true' split was defined as such (BC03 versus BC09)? The reason I am asking this is also because BC03 is a mixture of both primary and metastatic lymph node cells, while BC09 is only from primary resection (line 270). So, one could ask what if the 'true' split was made at primary versus metastatic level. Any clarification would be great.

3. Comments on the methods:

a. Central to the method is the bayesian framework of modeling conditional probabilities of observing alternate alleles, P(X_cg|.) using beta-binomial distribution. When there is no mutation, i.e. P(Zg = 0), the binomial probability parameter is a constant epsilon capturing errors in reverse transcription/sequencing/mapping. Whereas, in the case of mutation, i.e. P(Zg=1), the prior on the binomial probability is modeled using beta distribution. Figure 1B shows how the parameters of the beta distribution can be estimated empirically across the cells for any locus g. The derivations in the methods and the supplemental material are helpful for readers to follow the derivation and modeling framework. Please see my questions / comments below.

i. (Typos) In the supplemental material, when the five probabililities are listed for d^{g}_cc', please note that there are some missing accent marks. It would also be great if you could (re-) mention how P_g is exactly defined in this section. Along the same note: there are also missing accent marks in line 419 for P(X_c'g|.); (not critical) in line 421, maybe add Zc'g = 0 as well.

ii. Is epsilon also estimated empirically? (Or is this estimated during the maximum likelihood estimation?)

b. Please annotate Figure 2A more. In particular, how "overlaying" the scRNA-seq count information onto the mutation profile (Z) matrix - as is mentioned in the text (line 158) - is exactly done is not clear. Therefore, it is not entirely clear how the central (colorful, cells by mutation) matrix in Figure 2A is generated. In other words, how is the spike-in data constructed exactly? Also, what do the dotted lines in the DENDRO tree (in Figure 2A) represent?

c. Could you possibly elaborate on lines 484 and 485 (also for the general readers)? It is not clear to me what [(m-g)e + g(1-e)], for instance, exactly capture for reference alleles. A recommendation is to use different annotation for 'g' here representing genotype from 'g' elsewhere referring to a locus. What is the significance of 'g*' in line 485?

d. (SMART-seq vs other libraries) All the validation and implementation studies done in the paper make use of SMART-seq sc-RNA-seq library. This makes sense in that full length transcripts give better transcriptomic coverage over 3' or 5' libraries. Nonetheless, have the authors compared the performance for non-full length transcriptomic libraries?

e. (Github-related) The authors have provided a github repo for DENDRO and DENDROplan, which appears user-friendly. In the case of DENDROplan vignette, parameters 'kprob' and 'lprob' are explained, but (unless i missed this) please provide some additional information on the 'k' and 'subtype' parameters. Specifically, for example, how is Figure 3C dendrogram (in the vignette) with k=2, subtype=1 different from the example in 3.4.1 Example 1.

4. Other minor comments:

a. (Clarification) In "X/N matrix", is it either of the two matrices or are the elements in X normalized?

b. (Possible typo) Line 394 : Mutect2 does or does not account for splice junction? (Based on the language, 'not' is missing.)

c. Figure 2C shows that Z matrix based clustering performs better than X/N based clustering. So, the question for Figure 2B is how DENDRO performs compared to Z-matrix based clustering. (It would be better to show that as well/instead).

d. (Typo) In Figure 2 caption, for 2B, i believe it should read "Left panel: effect of mutation burden on fixed read depth" (and not "effect of read depth"). Similarly, "Right panel: effect of read depth on fixed mutation burden".

e. In Fig S2 caption, please briefly state what the parameters are and point the readers to the Supplemental Material for details. (These parameters are discussed only in the Supplemental material - and not in the main text.) Along this note, in the corresponding text in the supplemental material, what do H, M and L exactly refer to?

f. In Fig S11 caption, (not critical but) please mention what the rows and columns represent. In this and other similar heatmaps, please mention how the 'smoothing' of the expression is done (by labeling the color key or in caption)?

g. In line 235, please refer to Figure 3C (else, readers might still be looking at Figure 3B to visually affirm).

h. (Insignificant / non-critical) A few grammatical typos were noticed. For instance:

- In line 169, 'quantify' should be singularized ('quantifies').

- In line 157, missing "allele" (after alternative)

i. Users would be interested in knowing how long it takes to run the script, if you can provide any reference notes on that.

**Authors Response**

**Point-by-point responses to the reviewers’ comments:**

Reviewer #1: The manuscript describes an approach called Dendro for single-cell RNA-seq data. Dendro first characterizes the mutational profiles in each cell, then clusters cells into subclones from which it builds a clonal tree relationship. The relationship can then be used as the basis for further analysis of the transcriptomic aspect of the data. This approach is unique in that it requires no additional data and the case-studies shown are very motivating and would be of broad interest though especially to cancer researchers.

*Thanks for reviewing our paper and for your helpful comments! For your convenience, we have marked all the edited lines with a black bar on the left margin.*

Major Comments:

1. The term 'spike-in procedure' was quite misleading for me since spike-ins have very distinct meaning in transcriptomics. My understanding is that this is a simulation procedure where the structure is simulated rather than the counts themselves. I suggest the authors simply refer to this as a simulation and the explanation given is sufficient.

*We thought of this as in silico spiking in mutations to a real scRNA-seq data set, but agree that it is confusing. We have substituted all instances of “spike-in” by “simulation”.*

2. The sentence in line 58-60 regarding limitations to sequencing DNA and RNA in the same cell needs appropriate citations.

*We added four citations: two review papers on the limitation of joint DNA-RNA profiling and (2) two papers that profile DNA-RNA jointly but only restricted to limited number of cells (10 and 130 cells respectively).*

3. Equation on line 408, and explanation in line 411-412. If they are from the same clonal group, does that mean the numerator will be smaller than the denominator or just not as large as when they are from different clones? This could be explained more clearly.

*We agree that this is confusing. If the cells are from the same clonal group, the denominator indicating probability of two cells belonging to the same clone would be close to 1 and the distance between two cells becomes smaller. We have rewritten the sentence to make the point clearer.*

4. I appreciate the figure overviews, but some are difficult to follow through. Below are suggestions:

(i) Figure 1B: Is the top and middle representing both the true underlying data and the bottom is what is observed in the data as a result? Consider adding labels to indicate the distinction or in the legend.

*We apologize for the confusion. We agree that without detailed description, the method is hard to comprehend. As illustrated in panel 2 and 3, gene g is a bursty gene while g’ is a constitutive gene. Thus, even though we observe the same allele information between g and g’ in c1 and c2, it is more likely to observe extreme values in gene g (g is more bursty) and thus, we assign less weight in g (i.e. dg*

(ii) Figure 2A: I would label that the mutation profile is simulated and then indicate that bulk is input and then moderated by existing single-cell reference data.

*Thanks for your suggestion. We have added additional labels and legends to clarify Figure 2a.*

5. QC metrics to retain cells and genes need to be explicitly described. Both the criteria based on mutation, and any typical considerations regarding total number of reads or % mitochondrial reads.

*Thank you for pointing this out. We have added a subsection “Data preprocessing and quality control” in the Methods, which includes the details of QC criterion.*

6. Did the authors generate data for this paper on the B16 dataset? If so, where is the data deposited at? For data obtained from publicly available datasets, the GEO/SRA #'s should also be given explicitly.

*Yes, we generated the B16 dataset in this paper. Now, the B16 dataset is publicly available at NCBI GEO under accession number GSE139248. Other dataset we applied (but not generated) in the manuscript are also listed in the “Availability of data and materials” section.*

7. All datasets examined used the Smart-seq full-length protocol. Is it possible to use data from tag based reads (10X, Drop-seq) with Dendro? If so, it would be useful to see as a case-study, or if not then it would be helpful to have a statement on this in the discussion.

*We thank the reviewer for the comments. We indeed have tested DENDRO on multiple 10X data sets. However, due to the fact that (1) 10X and, in fact, any UMI-based data only profile the 3’ end of the transcripts and (2) sequencing depth is very shallow (<0.1X), we didn’t have much success in subclonal structure identification with DENDRO. As only one gastric cancer (unpublished) data set showed subclonal structure with high confidence (hypermutation in one subclone), we decided not to claim that DENDRO will work on 10X and Drop-seq based scRNA-seq analysis. To reflect this point, we added a statement in the Discussion.*

8. The software installed and ran as intended. The authors should also add example code on how to make the circular plots like in the paper as that will encourage more users of the package.

*Thanks for testing our software and the suggestion. We have updated our software accordingly. You can now check the new vignette with fan plots available at https://rawgit.com/zhouzilu/DENDRO/master/vignette/DENDRO_vignette.html .*

Minor Comments:

1. The statement ", that we modeled in DENDRO." In line 65 is a bit odd since DENDRO has not yet been introduced and this is still the background section.

2. Line 395 should say "does not".

3. Clusters on Figure 4b are not indicated as dashed lines.

4. Line 432, the rates, alpha and beta, are estimated empirically as what exactly?

*These are now fixed. Thanks for spotting them.*

Reviewer #2: The authors have developed a statistical framework, DENDRO, to identify genetically distinct subclones in tumor samples using single-cell (sc-) transcriptomics data. DENDRO accounts for technical noise (induced by errors in reverse transcription, sequencing and mapping) and expression level stochasticity (associated with single-cell level expression bursting) currently inherent in the single-cell transcriptomics library. Once the scRNA-seq-derived mutation profiling is done, DENDRO computes the divergence between pairs of single cells by modeling the conditional mutation frequency (per cell and locus) in a bayesian framework using beta-binomial model (few extensions on this discussed below). With this modeling framework, through simulation and spike-in analyses, the authors show that genetic divergence score is (i) orthogonal to the relative expression level and variation in total expression in genes, and (ii) improves with larger number of mutations profiled, among others. In addition, the authors also show that DENDRO significantly improves over other clustering approach such as hierarchical clustering based on mutation or expression matrix alone and identifies clones better than with scRNA-seq based expression data alone. The authors implemented DENDRO on three real datasets: (i) Implementation on Renal cell carcinoma data with known clonal identity was used as a validation study, with positive affirmation as mentioned above. (ii) subclonal expansion in mouse melanoma model in response to immunotherapy, showing DENDRO identified subclones showing diverging tumor mutational burden and predicted neoantigen load. (iii) primary and lymph-node metastasis in breast cancer samples, showing that DENDRO clusters can identify heterogeneity in otherwise previously mono-labeled breast cancer samples.

Overall the authors have mostly thoroughly explained their approach and model. The implementation of the model, thanks to the R-package, should help users of sc-RNA-seq get genomic mutation profile and clonal detection. The authors have shown that DENDRO performs relatively well with low read coverage and is capable of subclonality detection that are potentially missed by expression level data alone. However, please consider the following comments and questions.

*Thanks very much for reviewing our paper and for your constructive comments! For your convenience, we have marked all the edited lines with a black bar on the left margin.*

Some comments:

1. Inherently, none of the mutations in the non-coding elements in the genome are considered in this framework/model. While this is a limitation of the assay type itself (scRNA-seq) - and the DENDRO beta-binomial model, by design, only handles mutations along the transcriptome - how robust would the DENDRO clustering itself be to not using non-coding mutational data? In practice, if DENDRO clustering is in fact robust to this (not using non-coding mutational profile), it is also saying that those non-coding mutations do not largely contribute to estimating genetic divergence score, which we know is not necessarily the case. While this (transcriptome-based DENDRO vs 'whole genome mutational profile-based DENDRO') analysis is out of the scope of this paper, the question for the users now would be whether to do scDNA-seq based mutational profiling followed by clonal identification versus just using the scRNA-seq data and DENDRO (which would be cheaper but limited in not having non-exonic mutational profile). Any comment here is appreciated.

*We appreciate your concern and understand that DENDRO has this limitation for incorporating mutations in non-coding regions for clonal inference. To inform the reader, we have added this limitation in the Discussion section. This limitation is also shared by targeted sequencing and whole exome sequencing, currently the two most common methods for tumor genetic profiling. There are two reasons for such targeted methods: (1) Even limiting to the coding regions, we already detect plenty of mutations to infer clonal development. As there are more passenger mutations than driver mutations, and passenger mutations (especially synonymous mutations), in general, occur in both coding and non-coding regions. The tree inferred from coding region is a good estimate of the true underlining tumor evolution. But we agree that there will be exceptions. (2) Whole genome sequencing, especially at the single cell level, is still too costly to do on a large scale. In contrast, DENDRO gives an estimate of the DNA-based phylogeny and paired transcriptome information from scRNA-seq, which is a cost effective alternative. Thus, forsaking the noncoding variants through this experimental design allows us to still do meaningful analyses in a cost effective manner.*

2. My understanding is that the performance of clustering (with ARI score) depends on the prior establishment of 'true' tree. In the case of the first implementation (Renal cell carcinoma samples) the clustering was largely separating primary cells from metastatic cells (line 199). However, in the case of the third implementation (primary and lymph-node metastasis in breast cancer samples), DENDRO separates BC03 cells from BC09 cells - and not primary versus metastatic cells - with 100% accuracy. This is not to say the primary versus metastatic split is universal, but, is it not that the ARI is 100% in this case because the 'true' split was defined as such (BC03 versus BC09)? The reason I am asking this is also because BC03 is a mixture of both primary and metastatic lymph node cells, while BC09 is only from primary resection (line 270). So, one could ask what if the 'true' split was made at primary versus metastatic level. Any clarification would be great.

*This is a good question and indeed, we see that our description of the breast cancer analysis can be confusing. In the revision, we rewrote some parts of this section. Back to your point, unlike RCC, where all of the cells came from the same patient, the cells from BC came from different patients (patient BC03 and patient BC09). Inter-individual genetic distances far exceed intra-individual genetic distances, as shown in almost all single cell tumor studies (Patel et al. 2014 Science, Tirosh et al. 2016 Science, Navin et al. 2014 Genome Biology). As (1) most of the passenger mutations happen at random and (2) there are infinite potential mutation loci in a genome (3.2 billion base pair), observing two tumors from two patients have the same exact mutation profiles is very unlikely. However, as metastasis derived from primary site, in theory, the metastasis and primary site within the same patient share same ancestry and thus, have higher number of overlapped mutations. As a result, we expect cells from BC03 to separate from the cells from BC09.*

*In addition, metastasized cells from regional lymph nodes (BC) can be quite different from cells at distant metastasis (RCC). Lymph-node infiltrated tumor cells are at an intermediate stage (Naxerova et al. 2017 Science): (1) tumor cells from primary site are continuously infiltrating into the regional lymph node; (2) at the same time, early infiltrated tumor cells start to diverge from those remaining at the primary site. Thus, subclone mixture between primary and lymph node metastasis is expected, as observed in the BC analysis.*

3. Comments on the methods:

a. Central to the method is the bayesian framework of modeling conditional probabilities of observing alternate alleles, P(X_cg|.) using beta-binomial distribution. When there is no mutation, i.e. P(Zg = 0), the binomial probability parameter is a constant epsilon capturing errors in reverse transcription/sequencing/mapping. Whereas, in the case of mutation, i.e. P(Zg=1), the prior on the binomial probability is modeled using beta distribution. Figure 1B shows how the parameters of the beta distribution can be estimated empirically across the cells for any locus g. The derivations in the methods and the supplemental material are helpful for readers to follow the derivation and modeling framework. Please see my questions / comments below.

i. (Typos) In the supplemental material, when the five probabililities are listed for d^{g}_cc', please note that there are some missing accent marks. It would also be great if you could (re-) mention how P_g is exactly defined in this section. Along the same note: there are also missing accent marks in line 419 for P(X_c'g|.); (not critical) in line 421, maybe add Zc'g = 0 as well.

*Thanks for pointing these out. The typos are fixed*

ii. Is epsilon also estimated empirically? (Or is this estimated during the maximum likelihood estimation?)

*This is an important point. The value of epsilon is decided by prior knowledge based on sequencing machines. Here, for Illumina, epsilon is set to 0.001 (https://www.nature.com/articles/s41598-018-29325-6). We have added this point in the Methods section*.

b. Please annotate Figure 2A more. In particular, how "overlaying" the scRNA-seq count information onto the mutation profile (Z) matrix - as is mentioned in the text (line 158) - is exactly done is not clear. Therefore, it is not entirely clear how the central (colorful, cells by mutation) matrix in Figure 2A is generated. In other words, how is the spike-in data constructed exactly? Also, what do the dotted lines in the DENDRO tree (in Figure 2A) represent?

*Thanks for this comment and we apologize for the confusion. When generating the spike-in data set for this analysis, we did not utilize the expression scale information (high vs medium vs low expressed loci). As mentioned in the Method section, we adopt a scRNA-seq dataset from Deng et al as the reference, which has allele specific read counts for each variants detected (by cross two mice from different strains). When we overlap this single cell expression data on simulated mutation profile, for every locus we simulated, we sample from this reference and randomly assign one allele as mutated allele. We have added detailed simulation procedure in the Method section.*

*scRNA-seq data WES and bulk RNA-seq in Figure 2a belong to the pipeline of DENDROplan. We have modified Figure 2a according to this comment. When predicting the DENDRO clustering accuracy with DENDROplan, simulated RNA matrix was generated based on pioneer bulk RNA-seq. In another word, variants in high-expressed genes in bulk will be sampled from high-expressed variants loci pool in scRNA reference, while variants in low-expressed genes in bulk will be sampled from low-expressed variants loci pool in scRNA reference. Simulated RNA-seq from such scheme best mimic the real data and provide more confident estimation of DENDRO’s predicted clustering accuracy.*

*The dash lines in the DENDRO tree (Figure 2a) represent cluster cuts, and we agree this is confusing. We have modified Figure 2a to make the clusters easy to perceive.*

c. Could you possibly elaborate on lines 484 and 485 (also for the general readers)? It is not clear to me what [(m-g)e + g(1-e)], for instance, exactly capture for reference alleles. A recommendation is to use different annotation for 'g' here representing genotype from 'g' elsewhere referring to a locus. What is the significance of 'g*' in line 485?

*Thanks for your suggestion. We now denote genotype with “s”. According to your suggestion, we have also rewritten this paragraph for better clarity.*

d. (SMART-seq vs other libraries) All the validation and implementation studies done in the paper make use of SMART-seq sc-RNA-seq library. This makes sense in that full length transcripts give better transcriptomic coverage over 3' or 5' libraries. Nonetheless, have the authors compared the performance for non-full length transcriptomic libraries?

*We thank the reviewer for the comments. We indeed have tested DENDRO on multiple 10X data sets. However, due to the fact that (1) 10X and, in fact, any UMI-based data only profile the 3’ end of the transcripts and (2) sequencing depth is very shallow (<0.1X), we didn’t have much success in subclonal structure identification with DENDRO. As only one gastric cancer (unpublished) data set showed subclonal structure with high confidence (hypermutation in one subclone), we decided not to claim that DENDRO will work on 10X and Drop-seq based scRNA-seq analysis. To reflect this point, we added a statement in the Discussion.*

e. (Github-related) The authors have provided a github repo for DENDRO and DENDROplan, which appears user-friendly. In the case of DENDROplan vignette, parameters 'kprob' and 'lprob' are explained, but (unless i missed this) please provide some additional information on the 'k' and 'subtype' parameters. Specifically, for example, how is Figure 3C dendrogram (in the vignette) with k=2, subtype=1 different from the example in 3.4.1 Example 1.

*Thanks for testing our software and the suggestion. We actually had a typo in our vignette. K denotes number of subclones and subtype indicates different topological structures. Vignette’s Figure 3c should have been “k=4” as there are 4 subclones. The vignette on Github is fixed now, see https://rawgit.com/zhouzilu/DENDRO/master/vignette/DENDROplan_vignette.html .*

4. Other minor comments:

a. (Clarification) In "X/N matrix", is it either of the two matrices or are the elements in X normalized?

*Sorry for the confusion. X/N represents the variants allele frequency matrix, i.e. elements in X normalized by N. We have modified the format in the manuscript to “X/N” (format restriction on txt. See pdf version)*.

b. (Possible typo) Line 394 : Mutect2 does or does not account for splice junction? (Based on the language, 'not' is missing.)

*It is now fixed. Thank you for pointing it out.*

c. Figure 2C shows that Z matrix based clustering performs better than X/N based clustering. So, the question for Figure 2B is how DENDRO performs compared to Z-matrix based clustering. (It would be better to show that as well/instead).

*This is a great question. We have added the result on comparison with Z-matrix in Figure 2c. Here, to generate Z-matrix, we use maximum likelihood function based on GATK instruction. Due to high burstness of the original dataset and limited sequencing depth, we found that Z-matrix, on average, underperformed in all scenario. Comparing with PDX analysis, where thousands of mutation has been detected (even though most of them are false positive), it was not so surprise as Z-matrix outperform X/N.*

d. (Typo) In Figure 2 caption, for 2B, i believe it should read "Left panel: effect of mutation burden on fixed read depth" (and not "effect of read depth"). Similarly, "Right panel: effect of read depth on fixed mutation burden".

*These are now fixed.*

e. In Fig S2 caption, please briefly state what the parameters are and point the readers to the Supplemental Material for details. (These parameters are discussed only in the Supplemental material - and not in the main text.) Along this note, in the corresponding text in the supplemental material, what do H, M and L exactly refer to?

*This is great suggestion. We have added detailed notation in Figure S2 and explanation of “H, M and L” in the Supplementary Material.*

f. In Fig S11 caption, (not critical but) please mention what the rows and columns represent. In this and other similar heatmaps, please mention how the 'smoothing' of the expression is done (by labeling the color key or in caption)?

*Thank you for your comments. We have edited the figure and caption according to your suggestions. We added the details of “smoothing” method in the Supplementary Material.*

g. In line 235, please refer to Figure 3C (else, readers might still be looking at Figure 3B to visually affirm).

*These are now fixed. Thanks for spotting them.*

h. (Insignificant / non-critical) A few grammatical typos were noticed. For instance:

- In line 169, 'quantify' should be singularized ('quantifies').

- In line 157, missing "allele" (after alternative)

*These are also fixed.*

i. Users would be interested in knowing how long it takes to run the script, if you can provide any reference notes on that.

*Thank you for the suggestions. We have added a paragraph in the Method section explaining DENDRO’s computational complexity and computational time. Through optimization and vectorization, given a dataset of 500 cells with 2500 variants, the computation time is under 2 mins with a normal desktop (16GB of RAM, single thread). Analytically, the algorithm is of complexity O(N^2*G), where N is number of cells and G is number of variants.*

**Second round of review**

**Reviewer 1**

I appreciate all the authors responses and attentiveness in answering my previous questions and concerns.

Minor Comments:

1. Figure 1B&C still took me awhile to fully understand. I finally get it with help from the response. I’m including a suggested edit to the caption here. If the authors want to adapt at least part of it I think it would help future readers.

Suggestion:

b, c Statistical model for genetic divergence evaluation function. b. (top) Cell-level snapshots of the variant allele frequency (VAF) profiles for two genes with underlying differences in expression dynamics is shown. Gene 𝑔 is a bursty gene and 𝑔′ is a constitutive gene. (bottom) The stochasticity of gene expression is captured by the VAF distribution across all cells. c. Although the observed read counts from two potential cells (c1 and c2) in the population are identical, the full distribution of frequency profiles is accounted for when estimating the genetic divergence relationship between the two loci of these two cells.

2. The link to the vignette was given through https://rawgit.com, which says it will shutdown soon. The authors should link to the vignette on github another way. Note this does not affect any links in the manuscript.

**Reviewer 2**

Overall comment

The authors have, by and large, addressed many of the concerns and made suggested changes. The edits in Figure 1 have also made the approach easier to follow. Currently, my main comments (below) are around clarifications on the simulation study / Figure 2 (please see below).

--------------

(Questions regarding simulation study/Figure 2:)

To me, it is not 100% clear how the simulation study is done. Please see my questions in parenthesis below added in the 'Simulation analysis' excerpt from the Methods section (starting in line 470):

```

In our simulation analysis, we adopt a scRNA-seq dataset from Deng et al as the reference, which has allele specific read counts for each variants detected [43]. We overlapped this single cell expression data on simulated mutation profile.

(Lines 151-153 also states: "To get the matrix of alternative allele and total read counts for each mutation loci in each cell (𝑋 and 𝑁 , respectively), we overlay the mutation matrix onto the scRNA-seq data (See Methods for details)." However, it is not clear what "overlapping"/"overlaying" means/yields exactly.) For every simulated locus, we sampled a locus from this reference (this here refers to the allele-specific read counts data, right? - and not the scRNA-seq data.) and randomly assign one allele as mutated allele. We further added binomial noise to mimic sequencing error

(what are the binomial parameters used?). When analyzing DENDRO performance in terms of various number mutation site, number of cells, proportion of cells in each clade and proportion of mutations along each branch, we only take a subset of cells with similar cell types (similarity being defined as?).

```

2. While Figure 2A is slightly better to understand with added annotation, it could still use some brief additional clarification in the figure - and mostly in the figure caption, on how the simulation is done particularly.

3. (Minor) The authors have mentioned that they substituted all instances of “spike-in” by “simulation”. However, the changes are not made in figure 2B caption yet. Since the supplemental files are not provided in the revision, the changes need to be confirmed there as well.

--------------

Other minor comments:

1. (Regarding Z versus "X/N") Thanks for showing the accuracy with Z matrix in the simulation study. Please mention in the Figure 2 discussion briefly why Z matrix is potentially underperforming in the simulation study - as you have in the response.

2. Please change the title in the GEO repository to the new title.

3. For the BC data in the results section, please briefly mention (with citations) as to why a separation between BC03 and BC09 is expected - as you have discussed in the response.

4. (Minor note; no response required) In their comment on tag based sc-RNA sequencing, the authors mention "10X and, in fact, any UMI-based data only profile the 3’ end of the transcripts". The authors don't discuss 5-prime based sequencing platform. But this is still irrelevant considering the same concern (of only the transcript-ends being profiled) persists.

**Authors Response**

**Point-by-point responses to the reviewers’ comments:**

Reviewer #1: I appreciate all the authors responses and attentiveness in answering my previous questions and concerns.

*> Thanks for reviewing our paper and for your helpful comments!*

Minor Comments:

1. Figure 1B&C still took me awhile to fully understand. I finally get it with help from the response. I'm including a suggested edit to the caption here. If the authors want to adapt at least part of it I think it would help future readers.

Suggestion:

b, c Statistical model for genetic divergence evaluation function. b. (top) Cell-level snapshots of the variant allele frequency (VAF) profiles for two genes with underlying differences in expression dynamics is shown. Gene is a bursty gene and ′ is a constitutive gene. (bottom) The stochasticity of gene expression is captured by the VAF distribution across all cells. c. Although the observed read counts from two potential cells (c1 and c2) in the population are identical, the full distribution of frequency profiles is accounted for when estimating the genetic divergence relationship between the two loci of these two cells.

*> Thanks for your patience with this figure. We have built on your suggestion to revise the legend as follows: "b, c. Statistical model for genetic divergence evaluation function. b. (top) Cell-level snapshots of the variant allele frequency (VAF) profiles for two genes with underlying differences in expression dynamics is shown. Gene g is a bursty gene and g' is a constitutive gene. (bottom) The stochasticity of gene expression is captured by the VAF distribution across all cells. c. Although the observed read counts from two potential cells (c1 and c2) in the population are identical between the two loci, the genetic divergence computed from gene g is less than that computed from gene g' due to differences in transcriptional burstiness. DENDRO accounts for the full distribution of frequency profiles across cells when estimating the genetic divergence relationship between the two loci of these two cells. "*

2. The link to the vignette was given through https://rawgit.com, which says it will shutdown soon. The authors should link to the vignette on github another way. Note this does not affect any links in the manuscript.

*> Thanks for the reminder! The vignettes on github have been updated to raw.githack.*

Reviewer #2: I appreciate your responses and edits in the new revision. Please address some questions on the simulation study a little more. Thank you.

Reviewer #2: Overall comment

The authors have, by and large, addressed many of the concerns and made suggested changes. The edits in Figure 1 have also made the approach easier to follow. Currently, my main comments (below) are around clarifications on the simulation study / Figure 2 (please see below).

*> Thanks very much for reviewing our paper again and for your constructive comments!*

--------------

(Questions regarding simulation study/Figure 2:)

To me, it is not 100% clear how the simulation study is done. Please see my questions in parenthesis below added in the 'Simulation analysis' excerpt from the Methods section (starting in line 470):

```

In our simulation analysis, we adopt a scRNA-seq dataset from Deng et al as the reference, which has allele specific read counts for each variants detected [43]. We overlapped this single cell expression data on simulated mutation profile.

(Lines 151-153 also states: "To get the matrix of alternative allele and total read counts for each mutation loci in each cell ( and , respectively), we overlay the mutation matrix onto the scRNA-seq data (See Methods for details)." However, it is not clear what "overlapping"/"overlaying" means/yields exactly.) For every simulated locus, we sampled a locus from this reference (this here refers to the allele-specific read counts data, right? - and not the scRNA-seq data.) and randomly assign one allele as mutated allele. We further added binomial noise to mimic sequencing error

(what are the binomial parameters used?). When analyzing DENDRO performance in terms of various number mutation site, number of cells, proportion of cells in each clade and proportion of mutations along each branch, we only take a subset of cells with similar cell types (similarity being defined as?).

```

*> Thanks for the comments and we apologize for the confusion. To address your concern, we have rewritten the “Simulation analysis” excerpt in the Methods section. As mentioned in the revised manuscript, the simulation reference is a dataset from Deng et al., which, by crossing two mouse strains, obtained transcriptomic allele specific read counts for every SNPs in exonic regions in each cell using scRNA-seq. In this case, the Deng et al. data maintained the expression stochasticity in the read count matrices.*

*> “Overlapping/overlaying” means that for every simulated locus, we sampled a SNP from this reference. For cells with mutation at this locus, we randomly assigned one allele as mutated allele. For cells without mutation, we set the mutated allele counts as 0 and the total read counts as sum of the two alleles from the reference.*

*> The binomial noise parameter is set as p_ϵ=0.001, as suggested by Pfeiffer F et al. (Sci Rep 2018, [75] in the reference list).*

*> “Similar cell type” is well defined in the Deng et al. paper. Cells in early blastocyst, mid blastocyst and late blastocyst stages have indistinguishable expression profiles. Cells between 16-cell stages and blastocyst stages are dramatically different in transcriptome profile, which are used to show DENDRO’s robustness against heterogeneous expression profiles.*

*> We have added all the details in the revised Simulation analysis excerpt and hope this have addressed your concern.*

2. While Figure 2A is slightly better to understand with added annotation, it could still use some brief additional clarification in the figure - and mostly in the figure caption, on how the simulation is done particularly.

*> Thanks you for the suggestion. We have added brief explanation of the simulation process in the caption of Fig. 2a.*

3. (Minor) The authors have mentioned that they substituted all instances of “spike-in” by “simulation”. However, the changes are not made in figure 2B caption yet. Since the supplemental files are not provided in the revision, the changes need to be confirmed there as well.

*> We have double-checked and made sure all the “spike-in” has been replaced by “simulation” (both supplementary materials and supplementary figure legends).*

--------------

Other minor comments:

1. (Regarding Z versus "X/N") Thanks for showing the accuracy with Z matrix in the simulation study. Please mention in the Figure 2 discussion briefly why Z matrix is potentially underperforming in the simulation study - as you have in the response.

*> This has been addressed on Page 9 of the revised manuscript.*

2. Please change the title in the GEO repository to the new title.

*> This comment has been addressed. We updated the title in GEO to the new title suggested by the Editor. Please see https://www.ncbi.nlm.nih.gov/geo/query/acc.cgi?acc=GSE139248*

3. For the BC data in the results section, please briefly mention (with citations) as to why a separation between BC03 and BC09 is expected - as you have discussed in the response.

*> This comment has been addressed on Page 14 of the revised manuscript.*

4. (Minor note; no response required) In their comment on tag based sc-RNA sequencing, the authors mention "10X and, in fact, any UMI-based data only profile the 3’ end of the transcripts". The authors don't discuss 5-prime based sequencing platform. But this is still irrelevant considering the same concern (of only the transcript-ends being profiled) persists.

*> We now have also mentioned the 5-prime based sequencing in the discussion. Thanks for pointing this out.*
